# Gait Parameter Adjustments for Walking on a Treadmill at Preferred, Slower, and Faster Speeds in Older Adults with Down Syndrome

**DOI:** 10.1155/2012/782671

**Published:** 2012-05-30

**Authors:** Beth A. Smith, Masayoshi Kubo, Beverly D. Ulrich

**Affiliations:** ^1^Balance Disorders Laboratory, Departments of Neurology and Behavioral Neuroscience, Oregon Health and Science University, 505 NW 185th Avenue, Beaverton, OR 97006, USA; ^2^Department of Physical Therapy, Niigata University of Health and Welfare, Niigata 950-3198, Japan; ^3^Developmental Neuromotor Control Laboratory, School of Kinesiology, University of Michigan, Ann Arbor, MI 48109, USA

## Abstract

The combined effects of ligamentous laxity, hypotonia, and decrements associated with aging lead to stability-enhancing foot placement adaptations during routine overground walking at a younger age in adults with Down syndrome (DS) compared to their peers with typical development (TD). Our purpose here was to examine real-time adaptations in older adults with DS by testing their responses to walking on a treadmill at their preferred speed and at speeds slower and faster than preferred. We found that older adults with DS were able to adapt their gait to slower and faster than preferred treadmill speeds; however, they maintained their stability-enhancing foot placements at all speeds compared to their peers with TD. All adults adapted their gait patterns similarly in response to faster and slower than preferred treadmill-walking speeds. They increased stride frequency and stride length, maintained step width, and decreased percent stance as treadmill speed increased. Older adults with DS, however, adjusted their stride frequencies significantly less than their peers with TD. Our results show that older adults with DS have the capacity to adapt their gait parameters in response to different walking speeds while also supporting the need for intervention to increase gait stability.

## 1. Introduction

Persons with Down syndrome (DS) have lower tone and higher ligamentous laxity than their peers with typical development (TD), requiring them to find somewhat different solutions to control gait over their lifespan. For preadolescents with DS, merely increasing step width as compared to their peers with TD seems adequate to provide stability for walking overground at their self-selected speed [1, 2]. However, in response to the effects of aging, and at an earlier age than observed in the population with TD, adults with DS make additional changes to maintain gait stability while walking overground at their self-selected speed. Adults with DS aged 35–62 years walked slower, with shorter, wider strides and increased stance and double support periods than their age-matched peers with TD [3]. There are a number of factors known to affect gait patterns in older adults with TD that may contribute to the observed gait patterns in adults with DS, including neurophysiological changes associated with aging [4, 5], sedentary lifestyle [6], osteoarthritis [7], obesity [8], and Alzheimer's type dementia [9–14]. 

 While preadolescents with DS only need to make minimal adaptations (adjusting only step width) to their gait pattern to achieve stability while walking overground at their self-selected speed, they make many more adjustments when asked to walk on a treadmill [1, 2]. We found that preadolescents with DS shortened, widened, and increased stride frequency more than their peers with TD, at their preferred speed and speeds slower and faster than preferred [1, 2]. We attribute the increased adjustment of gait parameters for persons with DS during treadmill walking to the novelty and greater stability challenge presented by the treadmill [1, 2]. Our purpose here was to examine how older adults with DS adapt their gait when asked to walk on a treadmill at their preferred speed and at speeds slower and faster than preferred. The ability to adapt gait in response to changing environmental contexts is important in daily function. Further, it is possible that screening for adaptability, in a task like treadmill walking, might provide an early window on the gradual deterioration of gait function in everyday life activities. In our experimental design the treadmill increased the stability challenge beyond that of typical gait function, while slower and faster than preferred walking speeds pushed the participants' systems further out of their comfort zone and required gait adaptation. 

## 2. Method

### 2.1. Participants

 Twenty adults between 35 and 62 years of age participated in the study, 10 with DS and 10 with TD. Participants were matched for age (see Table 1). Adults with TD were healthy, lived independently, and were recruited through a university research volunteer website. Participants with DS were recruited from community support groups or through local supervised residences.

Of the 10 adults with DS, 6 lived in supervised residences while 4 lived with their parents. In terms of assistance needed for activities of daily living, 5 were able to independently bathe, dress, and feed themselves while 3 needed minimal and 2 needed moderate assistance with these activities. Five were able to shop independently while 2 required minimal assistance and 3 needed total assistance. Five of the adults with DS reported regular physical activity, mostly walking, 2–7 days per week, 10 minutes to 1 hour in duration. Eight had jobs consisting of light physical activity (e.g., housekeeping, packing boxes) from 3 to 30 hours per week.

 From their self and caregiver-reported health histories, the following health conditions were reported for the adults with DS (number reporting condition in parentheses): corrected vision deficits (8), obesity (6), heart murmur and/or valve repair surgery (5), occasional pain in hips and legs (6), corrected hearing deficits (5), hypothyroidism (5), hyperlipidemia (3), pes planus (4), dementia (2), high blood pressure (1), renal transplant (1), syncope (1), and seizures (1). For their overall health status, self-ratings were as follows: excellent (2), very good (4), good (1), okay (1), and declined to answer (2).

### 2.2. Procedures

All procedures were approved by the University of Michigan Institutional Review Board. When participants and their parents or caregivers came to the laboratory we explained all procedures and asked them to sign an assent or consent form, as appropriate. Next, participants changed into fitted shorts, a tank top, and removed shoes and socks. We attached markers (2.5 cm diameter) bilaterally to the skin at temporomandibular joint, acromion process, lateral humeral epicondyle, styloid process, greater trochanter, femoral condyle, 10 cm above the lateral malleolus, heel bony prominence, and third metatarsophalangeal joint. We placed EMG electrodes over the muscle bellies of the tibialis anterior, gastrocnemius, rectus femoris, and biceps femoris of the left leg. For the questions addressed in this paper EMG results will not be discussed.

We used a 6-camera Vicon Peak Motus real-time system (Vicon Motion Systems Centennial, CO) to collect reflective marker position data at 60 Hz as participants walked overground and on the treadmill. After 4–6 practice trials, participants walked at their preferred speed over a 5.3 meter GAITRite (CIR Systems, Inc., Havertown, PA) mat to the other side of the room. Each participant repeated this condition 4–6 times until we obtained 4 passes with all markers visible.

After overground data collection, the treadmill was moved into the calibrated space. From the overground trials we used GAITRite software to calculate average speed, which we used to set the treadmill speed for each individual performer. Participants were spotted while walking on a treadmill (Parker Brand) for 30-second data collection periods at 40%, 75%, and 110% of their comfortable overground walking speed. We defined the 75% speed on the treadmill as preferred pace [1, 2] and the 40% and 110% speeds as slower and faster than preferred. Participants practiced at each speed until they were able to walk comfortably (by their report) and maintain upright posture without holding onto the treadmill handrail. One participant with DS was afraid to walk at the fastest speed and so only completed the 40% and 75% treadmill speeds.

Anthropometric measurements were collected for calculation of dimensionless values. We measured weight (Healthometer beam scale), standing height (GPM anthropometer), sitting height, and length of the upper arm, arm, thigh, shank, and foot.

Participants with DS came to our laboratory for approximately 2 hours. We scheduled two sessions for adults with DS in order to keep each visit shorter and less stressful. During the first visit they walked overground followed by practice walking on the treadmill at their 75% speed. During the second visit we measured body segments and participants walked on the treadmill at 40%, 75%, and 110% speeds. Because adults with TD learn tasks faster and were more at ease with the testing conditions, they performed all tasks in one visit.

### 2.3. Data Analysis

We converted raw kinematic data to 3D data using PeakMotus software and a 6 Hz second-order Butterworth filter. We used a custom-written MATLab program (Mathworks Natick, MA) to identify initial foot contact and toe off events based on vertical acceleration of heel markers and horizontal accelerations of toe markers [15]. We used identified gait events, 3-D data, and anthropometric measurements to calculate dimensionless stride frequency, stride length, and step width values. Dimensionless values were used to account for leg length and leg length/trunk ratio differences [3]. For formulas and definitions of dimensionless parameters we used please see the appendix. Percent stance was calculated as the percent of each stride cycle between heel contact and toe off and thus included times when all or part of the foot was in contact with the ground.

We used SPSS (SPSS Inc., 233 S. Wacker Dr., Chicago, IL) version 19.0 for statistical testing. We set our alpha level of significance at 0.05. We used a multivariate analysis of variance (MANOVA) with repeated measures on speed and Bonferroni adjustments for multiple comparisons to test for a group effect, speed effect, and a group-by-speed interaction. Dependent variables were average dimensionless stride frequency, dimensionless stride length, dimensionless step width, and percent stance values for each participant at each speed.

## 3. Results

Table 2 presents absolute stride frequency, stride length, step width, and percent stance values by group and speed. The absolute values are provided to allow comparison to extant literature; however, we made our group comparisons based on dimensionless values to account for anthropometric differences between the populations.

Overall, the MANOVA demonstrated a significant group-by-speed interaction (Wilks' Lambda = 0.20, *F*[8,10] = 5.06, *P* = 0.01). There were also significant main effects of group (Wilks' Lambda = 0.20, *F*[4,14] = 13.85, *P* > 0.01) and speed (Wilks' Lambda= 0.01, *F*[8,10] = 88.65, *P* > 0.01), which are not interpreted due to the significant group-by-speed interaction.

**Figure 1:**
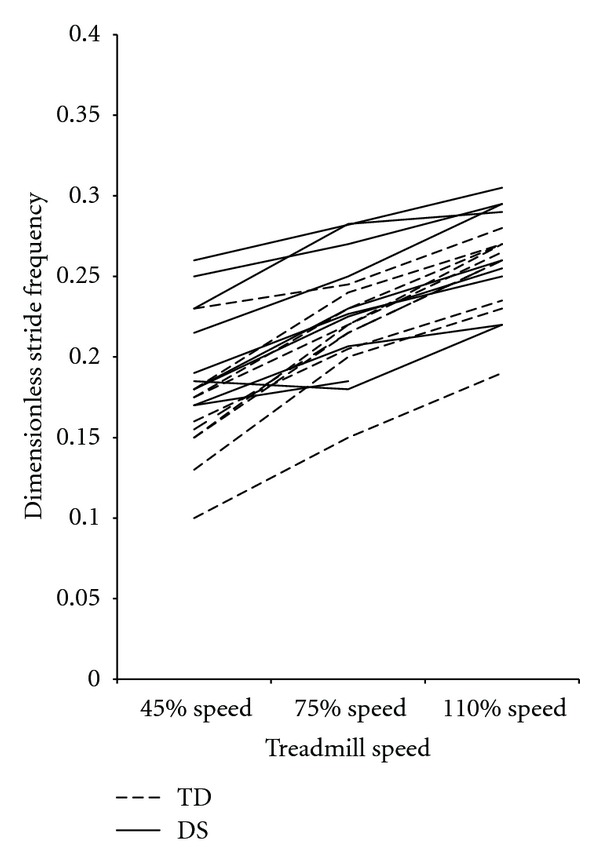
Mean dimensionless stride frequency values for each participant, by group and speed. Dimensionless stride frequency values increased as speed increased and were significantly different between groups at the 40% speed but not at the 75% speed or the 110% speed. DS: Down syndrome, TD: typical development.

Follow-up univariate analysis showed that the significant group-by-speed interaction was due to differences in adjustment of dimensionless stride frequency (Huynh-Feldt *F*(1.34,22.8) = 13.86, *P* > 0.01). Stride frequency increased from 40% to 75% to 110% speeds in both groups (pairwise comparisons *P* < 0.01 for all); however, there was less adjustment of stride frequencies at the slower speed by participants with DS as there was a significant group difference in dimensionless stride frequency at the 40% speed (*F*[1,17] = 8.74, *P* = 0.01) but not at the 75% (*F*[1,17] = 3.34, *P* = 0.09) or 110% (*F*[1,17] = 0.84, *P* = 0.37) speeds.  Figure 1 shows higher dimensionless stride frequency in the DS group compared to the TD group at the 40% speed but not at the 75% or 110% speeds.

**Figure 2:**
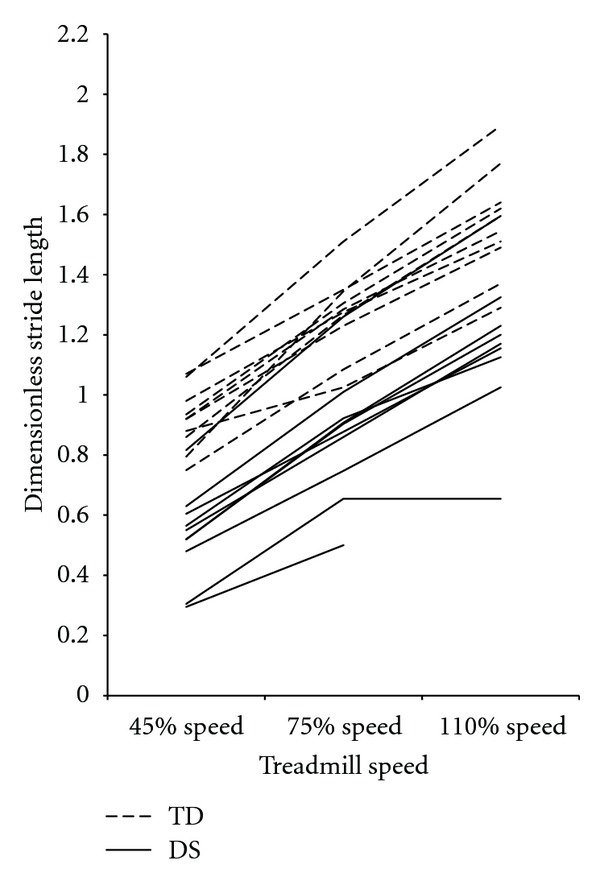
Mean dimensionless stride length values for each participant, by group and speed. Dimensionless stride length values increased with speed and were significantly different between groups at all speeds. DS: Down syndrome, TD: typical development.

**Figure 3:**
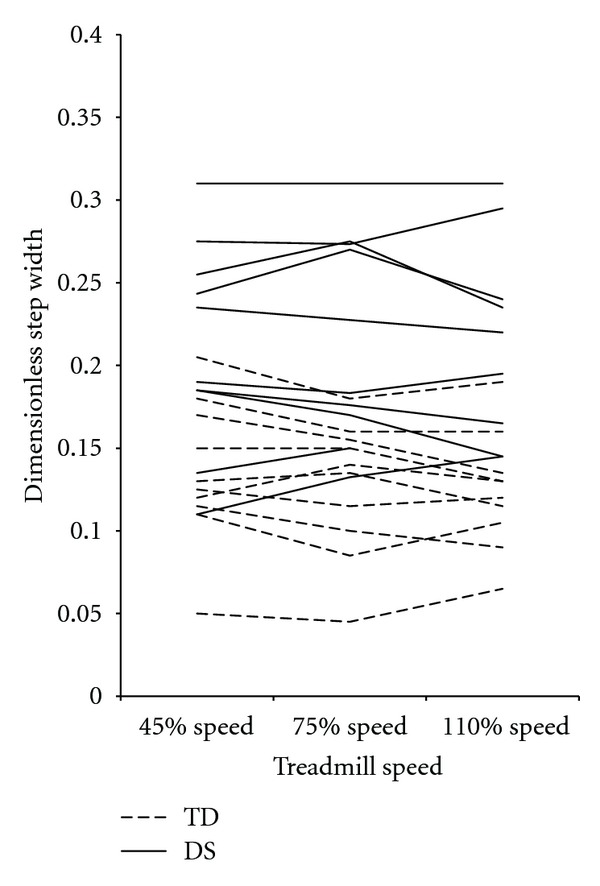
Mean dimensionless step width values for each participant, by group and speed. Dimensionless step width values did not change with speed and were significantly different between groups at all speeds. DS: Down syndrome, TD: typical development.

**Figure 4:**
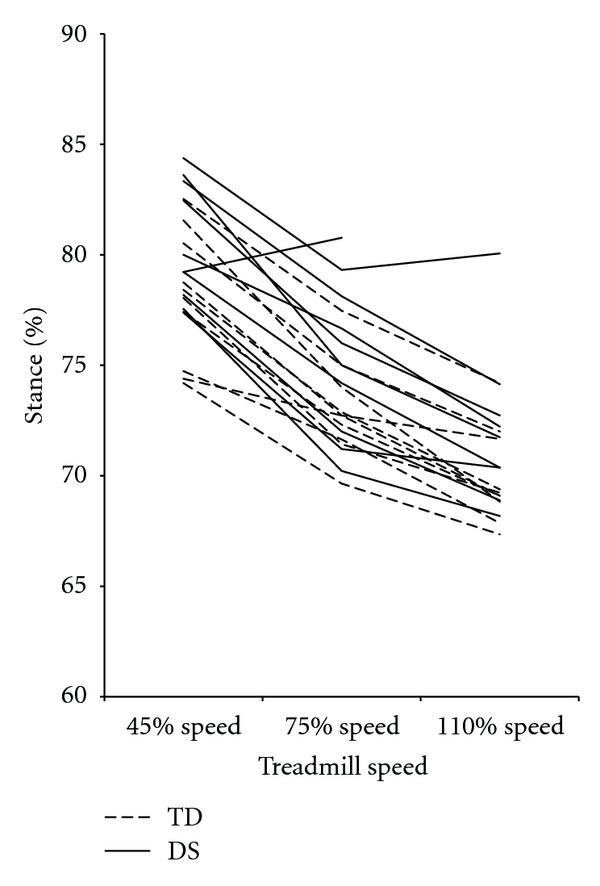
Mean percent stance values for each participant, by group and speed. Percent stance values decreased as speed increased and were not different between groups at any speed. DS: Down syndrome, TD: typical development.

Adjustments of stride length, step width, and percent stance were consistent between the DS and TD groups.  Figure 2 demonstrates that dimensionless stride lengths increased from the 40% to 75% to 110% speeds in both groups (pairwise comparisons *P* < 0.01 for all) and were shorter in the DS group at all speeds (40% = *F*[1,17] = 44.35, *P* > 0.01; 75% (*F*[1,17] = 21.93, *P* < 0.01; 110% (*F*[1,17] = 18.68, *P* < 0.01). As shown in Figure 3, dimensionless step widths, did not change by speed in either group (pairwise comparisons *P* > 0.05 for all) but were greater in the DS group at all speeds (40% = *F*[1,17] = 13.03, *P* = 0.01; 75% (*F*[1,17] = 12.94, *P* = 0.01; 110% (*F*[1,17] = 17.29, *P* = 0.01). Percent stance (Figure 4) decreased from the 40% to 75% to 110% speeds in both groups (pairwise comparisons *P* < 0.01 for all) and was not different between groups at any speed (40% = *F*[1,17] = 0.30, *P* = 0.59; 75% (*F*  [1,17] = 2.65, *P* = 0.12; 110% (*F*  [1,17] = 1.40, *P* = 0.25).

## 4. Discussion

 Overall, we found that older adults with DS in this sample were able to adapt their gait to slower, and faster than preferred treadmill speeds, by maintaining their stability-enhancing foot placements at all speeds. Previous work has shown that older adults with DS demonstrate slower preferred-speed overground gait with stability-enhancing adaptations of shorter strides, wider step widths, and increased stance and double support phases compared to adults with TD [3]. Here we found consistent stability-enhancing differences (shorter stride lengths and wider step widths) during treadmill walking for adults with DS at preferred, slower, and faster treadmill-walking speeds.

Both older adults with TD and DS adapted their gait patterns similarly in response to faster and slower than preferred treadmill-walking speeds. To adapt gait to the faster treadmill speed, all participants increased stride frequency and stride length, maintained step width, and decreased percent stance. To adapt gait to the slower treadmill speed, all participants decreased stride frequency and stride length, maintained step width, and increased percent stance. Older adults with DS, however, adjusted their stride frequencies significantly less than their peers with TD. In particular, adults with DS showed less decrease in stride frequency for the slower treadmill speed. Less ability to adjust stride frequency indicates some difficulty in adapting movement speed in adults with DS. Our results show they were more able to adjust foot placements (step width and stride length) than they were to adjust the movement speed of their stride (as reflected by stride frequency). This finding is consistent with previous reports of difficulty adjusting movement speed in persons with DS; however, it is also important to note that movement speed can be adapted following task practice [16–18].

Our findings are mostly consistent with previous research on treadmill walking in younger adults with DS (ages 19–44 years). Our results agree with those of Agiovlasitis and colleagues [19], who found that both groups (DS and TD) attained faster speeds by increasing step length and decreasing step time. Our results both support the use of stability-enhancing foot placements, although the precise findings differ slightly. In their study, adults with DS walked with faster, shorter-duration steps at all speeds and shorter steps at slow speeds, each of which would enhance stability by increasing the overall proportion of time spent with the foot in contact with the ground. Our studies do find conflicting results, however, for step width. In their study, adults with DS did not take wider steps than adults with TD (absolute or normalized to leg length). This may be due to their younger participants who appear to be more regularly physically active than ours or to slightly different procedures for normalization [19]. We consistently find wider step width for participants with DS compared to their peers with TD across the lifespan from new walkers to preadolescents to older adults, both for absolute values and values normalized to leg length [1–3, 20, 21]. We have not, however, tested participants between 10 and 35 years of age. Rigoldi and colleagues [22] reported wider step width in children (M 9.2 yrs, SD 2.5 yrs), teenagers (M 16.7 yrs, SD 3.2 yrs), and adults (M 37.5 yrs, SD 2.5 yrs) with DS during self-paced overground walking.

The gait adaptations we observed here in older adults with DS are very similar to what we found for preadolescents. In our previous study with the same treadmill walking measurements, preadolescents (ages 8–10 years) with DS and TD increased stride length and decreased step width as treadmill speed increased, although step width, even when normalized to leg length, remained wider in participants with DS. Preadolescents with DS demonstrated higher stride frequency than the group with TD and all participants decreased their stride frequency more at slower speeds [2]. Additionally, we used an escapement-driven inverted pendulum and spring model to measure global dimensionless stiffness and impulse values as a reflection of efficiency and stability of gait. We found higher levels of stiffness and impulse during treadmill walking for preadolescents with DS compared to their peers with TD, reflecting an increased need for stability and a less efficient gait pattern overall [1].

Although we did not measure stability and efficiency of gait directly in this study, our results do support the idea that older adults with DS demonstrate decreased stability and efficiency of gait. Adults with DS walk slower with shorter, wider strides than their peers with TD, consequently covering less ground with each stride likely using more energy to produce a gait pattern over a given distance. This pattern is robust, observed here consistently across different treadmill-walking speeds. Our results complement those of researchers who directly measured higher metabolic energy expenditure and lower cardiorespiratory function during treadmill walking in adults with DS [23, 24].

In previous work, we used nonlinear measures to analyze patterns of gait variability across the lifespan in persons with DS and found that older adults with DS demonstrated decreased adaptability of gait compared to their preadolescent peers with DS [25]. Our results here show that older adults with DS, despite less adaptability than their younger peers, still retain some capacity to adapt their gait parameters in response to different treadmill speeds. The older adults with DS in our study showed the ability to adapt their gait in response to changing environmental contexts, an important ability for daily function, and likely a feature that could be improved upon with appropriate intervention.

In addition to adaptability of gait, our results also support the need for intervention to increase gait stability as older adults with DS continue to demonstrate here, as well as previously [3], a need for increased walking stability earlier in life than adults with TD. This need for increased gait stability likely emerges from many factors. Age-related physiological changes may contribute [4, 5], as well as a sedentary lifestyle [6], osteoarthritis [7], and obesity [8]. Additionally, adults with DS experience a loss of oligodendrocytes in the basal ganglia [26], an area of the brain known to contribute to movement control. Abnormally high levels of oxidative stress [27] may induce a vulnerability for the very high levels of Alzheimer's type dementia (neurofibrillary tangles and plaques) observed in adults with DS over 40 years of age [9–11]. These physiologic and neural changes are experienced in addition to the inherent lifelong stability challenges of ligamentous laxity and hypotonia, with the culmination of these many effects likely contributing to less stable gait patterns in adults with DS.

Although researchers have acknowledged that there are multiple contributing factors to falls in adults with DS [28, 29], a causal relationship between decreased gait stability and adaptability and an increased risk of falls in adults with DS has not yet been shown. In fact, even the presence of increased fall risk in adults with DS has not been well documented. One group of researchers reported that adults with DS were less likely to fall than adults with epilepsy or autism [29]; however, their rate of falls was not compared to adults with TD. We previously reported that out of 14 adults with DS between the ages of 35 and 65 years who demonstrated high variability and decreased stability of gait, 6 reported a history of falls and 8 did not [21]. Prospective studies of fall risk in adults with DS, however, are lacking.

In addition to prospectively studying the risk of falls in adults with DS, future work is also necessary to determine how different factors relate to observed gait changes and mechanisms of falls in adults with DS. Our results show a continuum of gait performance in adults with DS, this is particularly apparent in the overlapping group values for dimensionless stride frequency (Figure 1) and dimensionless step width (Figure 3). Some adults with DS performed similarly to adults with TD while others did not. We did not run analyses on the relation between participants' specific characteristics and the dependent variables because our sample was too small to allow meaningful correlations. In the future, however, it is important to determine the weighted contribution of health status and lifestyle factors such as dementia, obesity, and inactivity to efficiency of gait patterns, falls, and quality of life. Once the relationship among factors is defined, appropriate (and likely multifactorial) interventions can be designed and tested in an effort to positively affect the health, mobility status, and quality of life of adults with DS. Although they likely will not be able to adapt to the same degree as preadolescents with DS as the challenges they face are greater, our results here support that adults with DS could likely improve and maximize the efficiency, stability, and adaptability of their gait with appropriate intervention.

## Figures and Tables

**Table 1:** Anthropometric measurements for adults with Down syndrome (DS) and typical development (TD), mean (standard deviation).

Group	Height (m)	Weight (kg)	Age (years)
DS adults	1.51 (0.08)	74.9 (22.9)	43.9 (8.7)
TD adults	1.59 (0.05)	64.5 (15.0)	43.6 (6.9)

**Table 2:** Absolute treadmill gait variables for adults with Down syndrome (DS) and typical development (TD), mean (standard deviation).

Group	Speed	Treadmill speed (m/s)	Step width (m)	Stride length (m)	Stride frequency (strides/s)	Percent stance (%)
DS adults	40%	0.28 (0.09)	0.14 (0.06)	0.37 (0.13)	0.77 (0.12)	80.6 (3.7)
	75%	0.54 (0.15)	0.15 (0.06)	0.60 (0.16)	0.91 (0.16)	75.6 (4.1)
	110%	0.80 (0.20)	0.15 (0.06)	0.79 (0.19)	1.01 (0.12)	71.9 (3.8)

TD adults	40%	0.39 (0.07)	0.10 (0.03)	0.68 (0.09)	0.59 (0.13)	77.5 (3.6)
	75%	0.73 (0.12)	0.09 (0.03)	0.94 (0.10)	0.78 (0.10)	73.2 (2.2)
	110%	1.07 (0.18)	0.09 (0.02)	1.17 (0.12)	0.91 (0.11)	69.4 (1.9)

## Appendix

Formulas for normalization to dimensionless values are

(A.1) $$\begin{array}{c} {\hat{w}}_{\text{STEP}}=\frac{w_{\text{STEP}}}{l_{O}}, \end{array}$$

(A.2) $$\begin{array}{c} {\hat{l}}_{\text{STRIDE}}=\frac{l_{\text{STRIDE}}}{l_{O}}, \end{array}$$

(A.3) $$\begin{array}{c} {\hat{f}}_{\text{STRIDE}}=\frac{f_{\text{STRIDE}}}{\sqrt{g_{}/l_{O}}}, \end{array}$$

where ${\hat{w}}_{\text{STEP}}$ (step width), ${\hat{l}}_{\text{STRIDE}}$ (stride length), and ${\hat{f}}_{\text{STRIDE}}$ (stride frequency) are the converted gait variables, **l**_*O*_ is leg length (sum of thigh length and shank length), and **g** is acceleration due to gravity.
